# Using a Classifier Fusion Strategy to Identify Anti-angiogenic Peptides

**DOI:** 10.1038/s41598-018-32443-w

**Published:** 2018-09-14

**Authors:** Lina Zhang, Runtao Yang, Chengjin Zhang

**Affiliations:** School of Mechanical, Electrical and Information Engineering, Shandong University at Weihai, Weihai, 264209 China

## Abstract

Anti-angiogenic peptides perform distinct physiological functions and potential therapies for angiogenesis-related diseases. Accurate identification of anti-angiogenic peptides may provide significant clues to understand the essential angiogenic homeostasis within tissues and develop antineoplastic therapies. In this study, an ensemble predictor is proposed for anti-angiogenic peptide prediction by fusing an individual classifier with the best sensitivity and another individual one with the best specificity. We investigate predictive capabilities of various feature spaces with respect to the corresponding optimal individual classifiers and ensemble classifiers. The accuracy and Matthew’s Correlation Coefficient (MCC) of the ensemble classifier trained by Bi-profile Bayes (BpB) features are 0.822 and 0.649, respectively, which represents the highest prediction results among the investigated prediction models. Discriminative features are obtained from BpB using the Relief algorithm followed by the Incremental Feature Selection (IFS) method. The sensitivity, specificity, accuracy, and MCC of the ensemble classifier trained by the discriminative features reach up to 0.776, 0.888, 0.832, and 0.668, respectively. Experimental results indicate that the proposed method is far superior to the previous study for anti-angiogenic peptide prediction.

## Introduction

Angiogenesis is a process of new blood vessel formations^1^, which involves multiple biological behaviors including endothelial cell proliferation, migration, apoptosis, cell-cell and cell-matrix adhesion^2^. It contributes to vascular remodeling and maturation^3^. Angiogenesis is tightly regulated by stimulators and inhibitors^4^. Appropriate balance between stimulators and inhibitors plays a pivotal function in maintaining and regulating angiogenesis, which often involves embryonic development, wound healing, menstrual cycle, and hair cycle^2^. Disruption of such an equilibrium is often associated with pathological processes^5,6^, including heart diseases, stroke, diabetes, blindness^2^, proliferative diabetic retinopathy, and atherosclerosis^7^. Especially, abundant evidence has indicated that imbalanced angiogenesis is involved in cancer progression^8,9^, due to the fact that the newly formed tumor vasculature provides stable blood supply for the growing tumor mass and eventually disseminates tumor cells that have escaped from the primary tumor^10^.

Angiogenesis inhibitors are needed to down-regulate the progression of angiogenesis, which would contribute to the development of therapeutic treatments for these angiogenesis-related diseases^11^. Previous studies have indicated that anti-angiogenic proteins or polypeptides can inhibit the angiogenesis process and have been applied in the therapies of cancers and other diseases^12^. However, most of anti-angiogenic proteins are large and complex, and they would cause some serious side effects^9,13^. In contrast to proteins and polypeptides, anti-angiogenic peptides have advantages for therapeutic application, in terms of their small size, lack of toxicity, lower immune reaction to the host system, higher solubility in water, higher stability, receptivity to chemical modification, and increased bio-availability^2^. In addition, they have a better ability to target and penetrate tissues^14^. Therefore, anti-angiogenic peptides have been shown as promising therapies for tumors and other angiogenesis-related diseases^15–17^.

Several anti-angiogenic peptide candidates which are currently in clinical trials are showing promising results^9,18^. For example, YSNS, a cyclized anti-angiogenic peptide, has been demonstrated to inhibit the capillary network formation in vivo and limit tumor growth in the small cell lung cancer^19^. KV11, a 12-mer peptide, has an ability to suppress tumor growth and tumor microvasculature in breast cancer xenografts^20^. Anti-angiogenic SPARC peptides have been investigated to inhibit progression of neuroblastoma tumors^21^. In view of the physiological functions and potential therapeutic purposes in organisms, identification of anti-angiogenic peptides may not only contribute to better fundamental understanding of the essential angiogenic homeostasis within tissues^22^. but also have significant implications for development of antineoplastic therapies^6^.

There are some computational and experimental methodologies to identify anti-angiogenic peptides. Based on the protein basic local alignment search tool (BLAST), searching the conserved domains of angiogenesis-associated proteins existing in the proteome is a common computational method to identify the putative anti-angiogenic peptides^23^. Homology modeling is another computational technique where the structure of an anti-angiogenic peptide is determined by comparison to a high-resolution structure or structures with sequence homology^9^. However, these two methods have a critical shortcoming that they can’t work when there are no homology sequences existing in the proteome. Computational screening via docking is a viable method of peptide discovery^9^. However, its complexity leads to a prohibitively expensive cost. Molecular dynamics (MD) is a computational simulation technique to identify the anti-angiogenic peptides, but the high computational cost hinders the process of MD^9^. In addition, experimental identification of anti-angiogenic peptides relies on an empirical process^4^, which is both labor intensive and time consuming^22^.

Recently, machine learning methods have been potential tools and have achieved promising results for identifying protein attributes. Ettayapuram Ramaprasad AS *et al*.^24^ developed a support vector machine (SVM)-based predictor to identify anti-angiogenic peptides, using various features extracted from peptide sequences including Binary Profile Patterns (BPP), Amino Acid Composition (AAC), and Dipeptide Compositions (DPC). The accuracy and Matthew’s Correlation Coefficient (MCC) of the method are 0.748 and 0.500, respectively. The prediction performance is acceptable, but there still exist the following shortcomings. (1) No feature selection technique was employed by the predictor proposed in the existing method^24^, which would lead to dimension disaster and poor performance^25^. Feature selection has the ability to get rid of redundancy information or noise and decrease model complexity^26^. (2) The method^24^ was based on an individual classifier which could have its own inherent defects^27^. It is generally accepted that the ensemble predictor integrating multiple basic classifiers of diverse learning policies (or diversely trained) is superior in carrying out statistics, calculation, and characterization analysis compared to its base classifiers^27^. Therefore, ensemble methods have been suggested as the promising measures for protein classification problems^28^.

**Figure 1:**
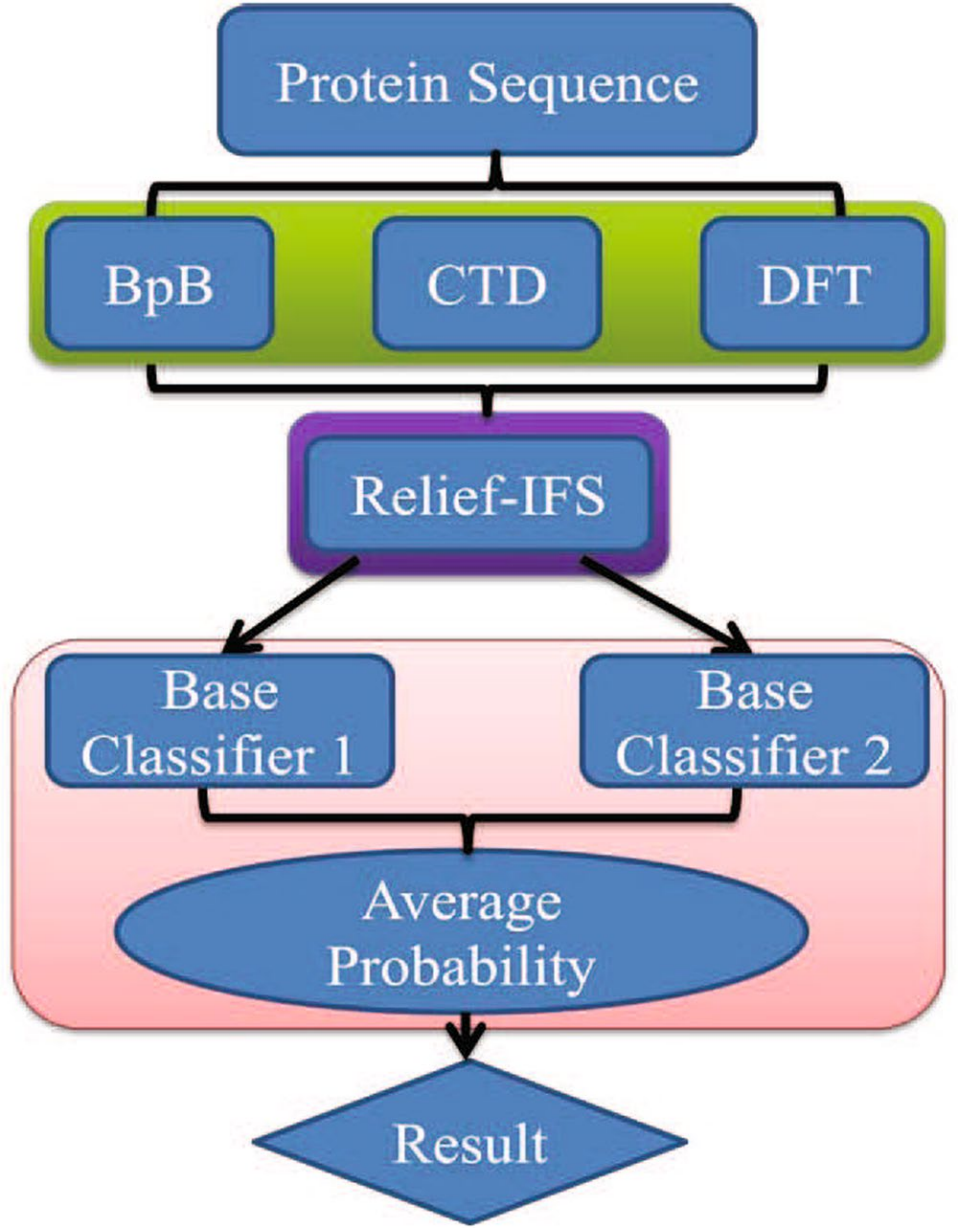
The construction process of the proposed anti-angiogenic peptide prediction model.

In view of the above shortcomings, a classifier fusion method as illustrated in Fig. 1 is proposed in this paper to promote the ability to predict anti-angiogenic peptides. We investigate predictive capabilities of various feature spaces including CTD (Composition, Transition and Distribution), BpB (Bi-profile Bayes), and DFT (Discrete Fourier Transform). These features are all related with the properties of the target peptides. To decrease the complexity of computation, the relevance of features and categories is assessed by Relief algorithm, and then IFS (Incremental Feature Selection) method is applied to capture a set of important features. Several individual classifiers are separately adopted to construct anti-angiogenic peptide prediction models. To achieve a better prediction accuracy, the classifier with the best sensitivity and the classifier with the best specificity are selected as the base classifiers. The final output of the prediction model is equal to the average probability for a given sample to be an anti-angiogenic peptide predicted by the base classifiers. 10-fold cross validation is carried out to verify the effectiveness of the prediction model. Simulation results show that the sensitivity, specificity, accuracy, and MCC of the proposed method reach up to 0.776, 0.888, 0.832, and 0.668, respectively, higher than those of the existing method^24^. The comparison results indicate that the proposed method is a promising tool for identifying anti-angiogenic peptides.

## Results and Discussion

### Performance of Various Feature Spaces on Different Individual Classifiers

**Figure 2:**
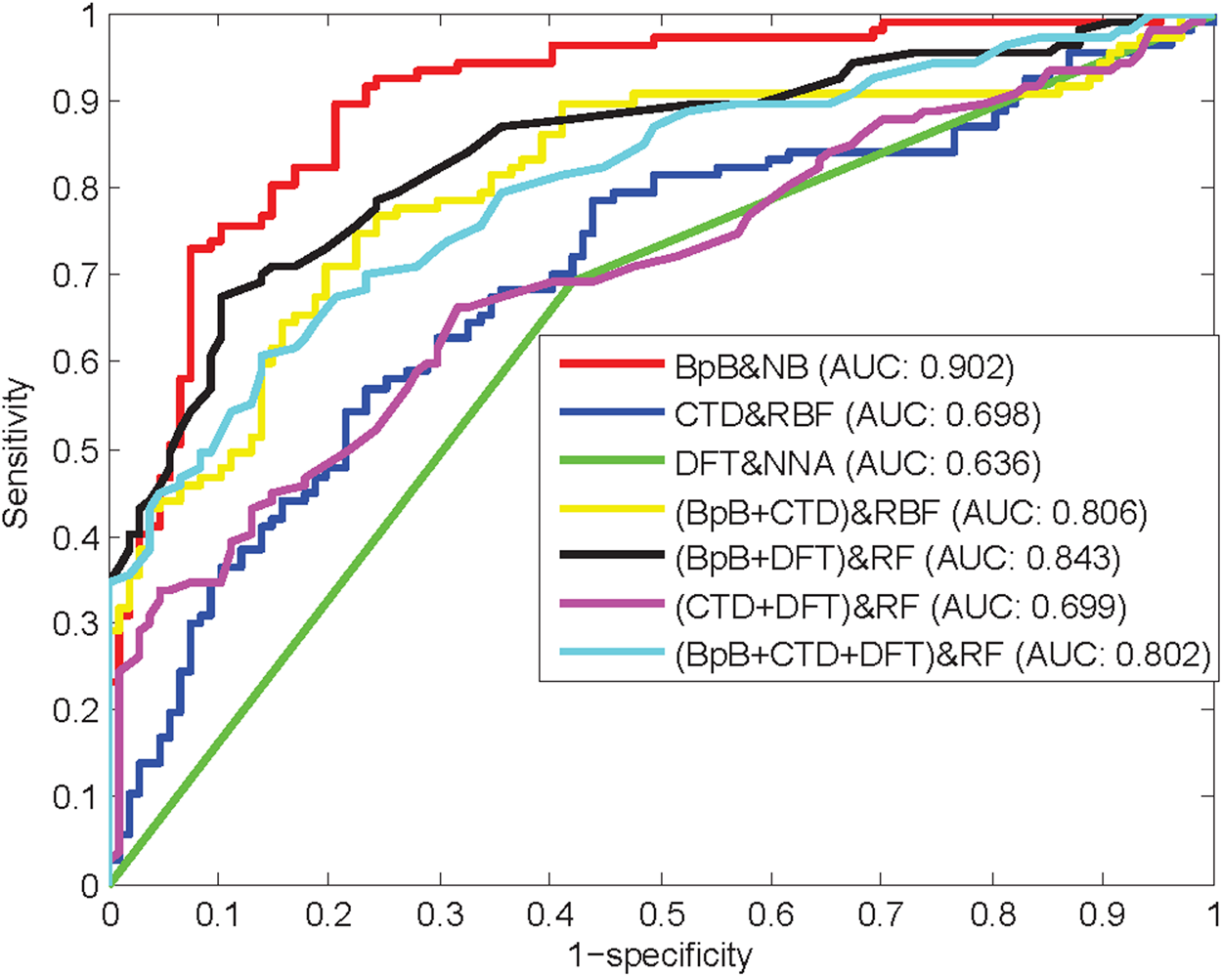
ROC curves of various feature spaces with respect to the corresponding optimal individual classifiers.

To investigate the optimal individual classifiers for different feature types, we evaluate the impact of various features on the performance of multiple individual classifiers. The prediction results of various feature spaces with respect to the corresponding optimal classifiers are given in Table 1. Figure 2 illustrates the receiver operating characteristic (ROC) curves of various feature spaces with respect to the corresponding optimal individual classifiers. As listed in Table 1, the prediction accuracy of various feature spaces with respect to the corresponding optimal classifiers is in the range of 0.636 to 0.804, indicating an ideal prediction effect for anti-angiogenic peptides. As shown in Fig. 2, the accuracy, MCC, and area under the ROC curve (AUC) of BpB is 0.804, 0.626, and 0.902, respectively, which represents the highest prediction results among the various feature spaces. These results demonstrate that statistical differences about the position-specific amino acid composition at the N-terminal region and C-terminal region are relatively discriminative in anti-angiogenic peptide identification, which is in accordance with research results in the previous study^24^.

**Table 1:** Prediction performance of various feature spaces with respect to the corresponding optimal individual classifiers.

Feature Space	Optimal Classifier	Sn	Sp	Acc	MCC	AUC
BpB	NB	0.682	0.925	0.804	0.626	0.902
CTD	RBFNetwork	0.551	0.766	0.659	0.325	0.698
DFT	NNA	0.692	0.579	0.636	0.273	0.636
BpB + CTD	RBFNetwork	0.701	0.804	0.752	0.507	0.806
BpB + DFT	RF	0.710	0.850	0.780	0.566	0.843
CTD + DFT	RF	0.664	0.682	0.673	0.346	0.699
BpB + CTD + DFT	RF	0.673	0.794	0.734	0.471	0.802

In addition, the optimal classifiers for different individual feature types are totally different (i.e., Naïve Bayes (NB) for BpB, Radial Basis Function Network (RBFNetwork) for CTD, and Nearest Neighbor Algorithm (NNA) for DFT). Except that the optimal classifier for BpB + CTD is identical to that for CTD, the optimal classifiers for hybrid feature spaces are totally different from those for their component feature types. These results show that an individual classifier is good at dealing with data classification with specific feature distribution. Except for BpB + CTD, other hybrid feature spaces have the identical optimal classifier, i.e., Random Forest (RF), demonstrating that RF is remarkable on managing data classification with complicated structure. In addition, except CTD + DFT, the accuracy values of hybrid feature spaces are not better than those of individual feature types. These results indicate that much redundant information may exist in hybrid feature spaces, which would deteriorate prediction performance in anti-angiogenic peptide prediction.

### Performance of Various Feature Spaces on Ensemble Classifiers

**Figure 3:**
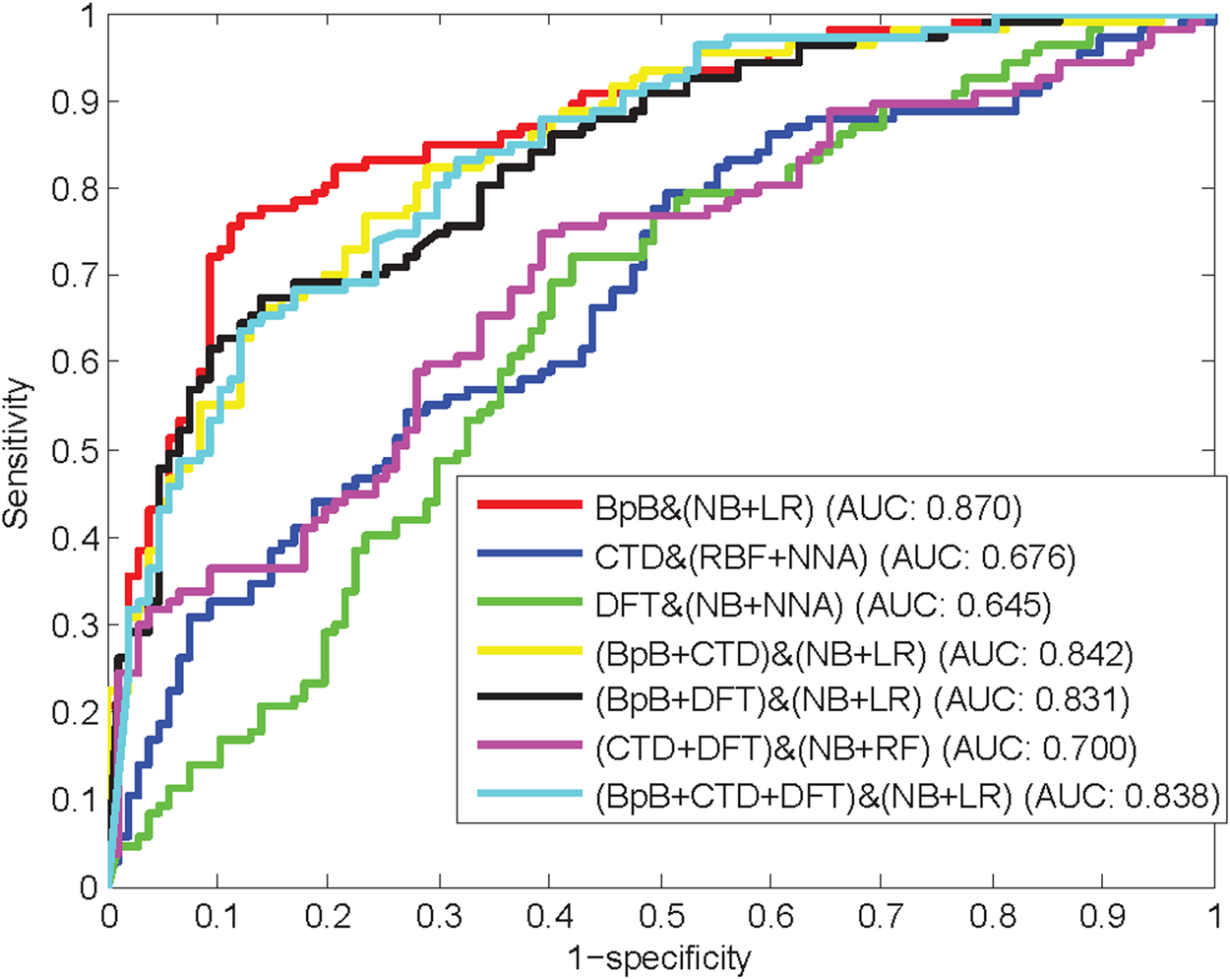
ROC curves of various feature spaces with respect to the corresponding optimal ensemble classifiers.

To investigate the best ensemble classifiers with respect to different feature types, we first examine the prediction performance of various features on multiple individual classifiers. Then, the ensemble classifier is determined by combining an individual classifier with a better sensitivity and another one with a better specificity. Table 2 shows the prediction results of various feature spaces with respect to the corresponding optimal ensemble classifiers. The ROC curves of various feature spaces with respect to the corresponding optimal ensemble classifiers are depicted in Fig. 3. From Table 2, for various feature spaces, the corresponding ensemble classifiers are not identical. However, except CTD, the ensemble classifiers for other feature spaces all have an NB classifier, indicating that an NB classifier can predict negative samples better than other individual classifiers. For half of different feature spaces, NB + LR (Logistic Regression) is the optimal ensemble classifier to identify anti-angiogenic peptides. Therefore, to verify the effectiveness of the ensemble method, the individual performance of NB classifier and LR classifier on the feature spaces with which the ensemble classifier NB + LR achieves best performance is separately given in Tables 3 and 4.

**Table 2:** Prediction performance of various feature spaces with respect to the corresponding optimal ensemble classifiers.

Feature Space	Optimal Classifier	Sn	Sp	Acc	MCC	AUC
BpB	NB + LR	0.766	0.879	0.822	0.649	0.870
CTD	RBFNetwork + NNA	0.617	0.57	0.593	0.187	0.676
DFT	NB + NNA	0.701	0.579	0.64	0.282	0.645
BpB + CTD	NB + LR	0.794	0.72	0.757	0.515	0.842
BpB + DFT	NB + LR	0.748	0.701	0.724	0.449	0.831
CTD + DFT	NB + RF	0.542	0.72	0.631	0.266	0.700
BpB + CTD + DFT	NB + LR	0.748	0.738	0.743	0.486	0.838

**Table 3:** The individual performance of NB classifier on different feature spaces.

Feature Space	Classifier	Sn	Sp	Acc	MCC	AUC
BpB	NB	0.682	0.925	0.804	0.626	0.902
BpB + CTD	NB	0.626	0.832	0.734	0.478	0.729
BpB + DFT	NB	0.570	0.804	0.687	0.384	0.704
BpB + CTD + DFT	NB	0.589	0.841	0.715	0.444	0.715

**Table 4:** The individual performance of LR classifier on different feature spaces.

Feature Space	Classifier	Sn	Sp	Acc	MCC	AUC
BpB	LR	0.785	0.748	0.766	0.533	0.766
BpB + CTD	LR	0.757	0.720	0.738	0.477	0.782
BpB + DFT	LR	0.738	0.682	0.710	0.421	0.710
BpB + CTD + DFT	LR	0.748	0.710	0.729	0.458	0.729

As shown in Table 3, there is a big difference between Sn and Sp achieved by NB classifier on different feature spaces. As shown in Table 4, although LR classifier achieves a much balanced Sn and Sp on different feature spaces, the Accs are not satisfactory. Compared with the NB classifier and LR classifier, the ensemble classifier NB + LR as given in Table 2 achieves a much better prediction performance on the corresponding feature spaces.

From Tables 1 and 2, hybrid features on the ensemble classifiers do not outperform the corresponding component individual feature types due to the redundant information in the hybrid features. The accuracy of BpB on the ensemble classifier is improved from 0.804 to 0.822. DFT, BpB + CTD, and BpB + CTD + DFT are all the same case with BpB on the corresponding ensemble classifiers. These comparison results reveal that an ensemble classifier can effectively improve prediction performance. However, there are exceptions for other feature spaces whose performance on the ensemble classier is worse than that on the optimal individual classier. In general, an ensemble classifier that integrates multiple basic classifiers of diverse learning policies (or diversely trained) can achieve better prediction performance than its component classifiers for protein attribute predictions^28^. These exceptions may be due to lack of diversity in learning policies of the component individual classifiers. The accuracy and MCC of the ensemble classifier trained by Bi-profile Bayes (BpB) features are 0.822 and 0.649, respectively, which represents the highest prediction results among the investigated prediction models using various feature spaces with different classifiers. In addition, from Fig. 3, BpB with the optimal ensemble classifier of NB and LR yields the best AUC of 0.870. Therefore, this study employs BpB with NB + LR to construct the final prediction model.

### Feature Selection Results and Corresponding Analysis

**Figure 4:**
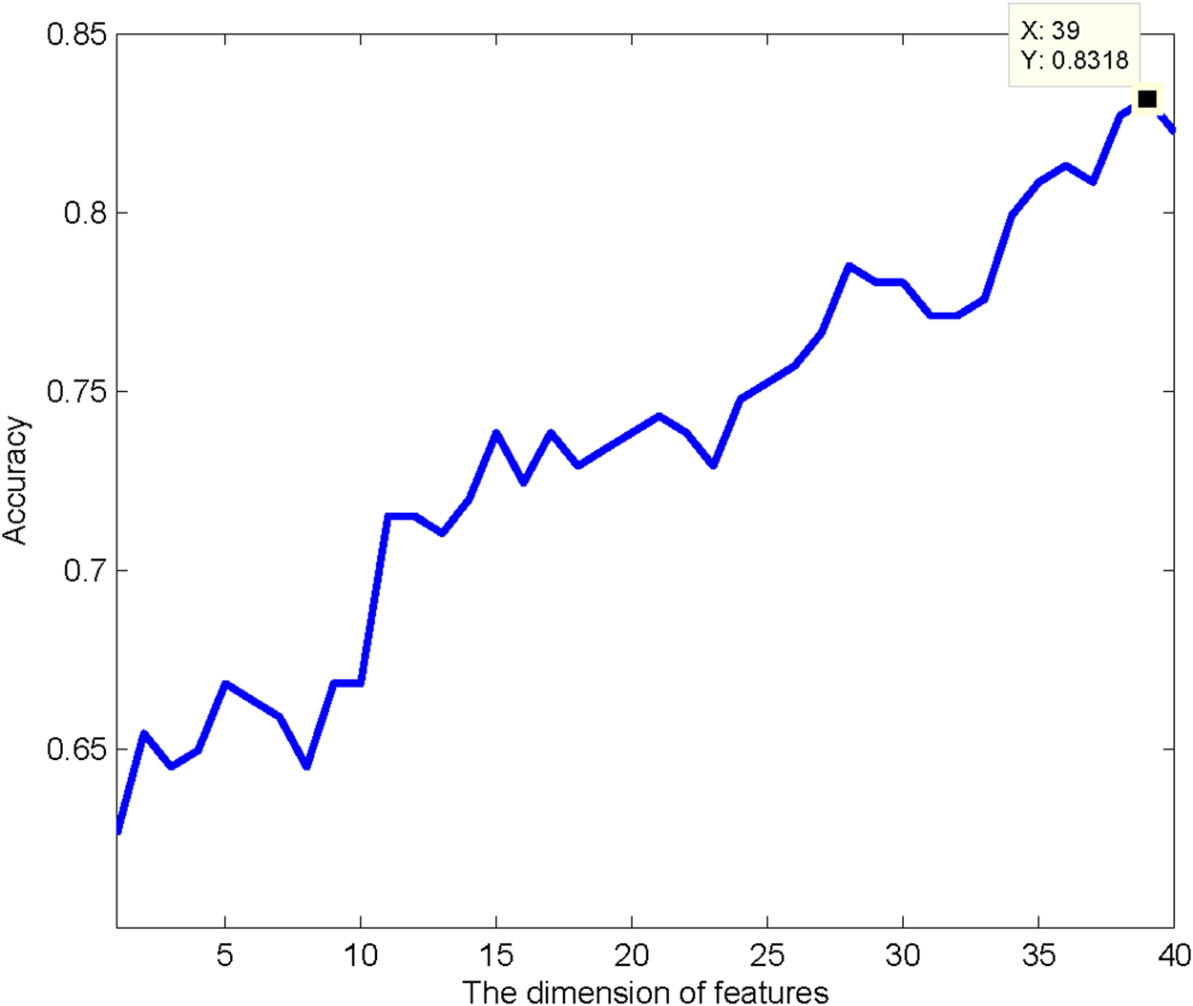
The IFS curve: the accuracy of the prediction model trained by different feature subsets.

The features extracted from the BpB method are sorted according to the weights from highest to lowest given by the Relief algorithm. As provided in Table S1, the feature with a higher ranking suggests that its ability to identify anti-angiogenic peptides is more powerful. Based on the feature ranking, the IFS method is implemented using the ensemble classifier NB + LR. Table S2 shows the detailed prediction results of the prediction model at each iteration based on 10-fold cross validation. As given in Fig. 4, the IFS curve that displays the accuracy of the prediction model at each iteration reaches a peak value when the prediction model is built by the first 39 features in Table S1. Thus, the first 39 features in Table S1 are selected to constitute the optimal feature subset for anti-angiogenic peptide prediction.

**Figure 5:**
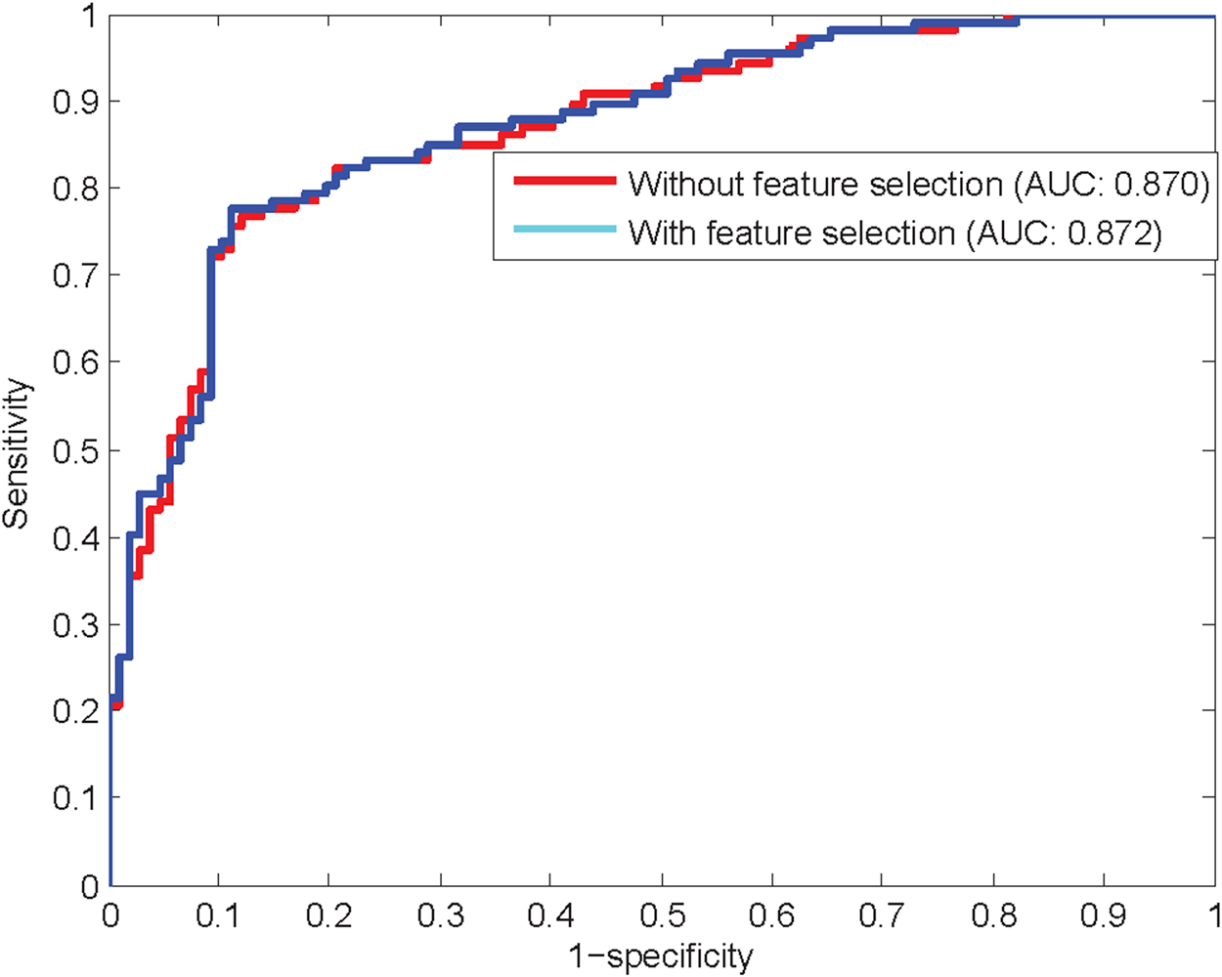
ROC curves with the proposed feature selection method or not.

To analyze the effectiveness of the proposed feature selection method, using the ensemble classifier NB + LR, the prediction models with and without the proposed feature selection method are separately constructed. As shown in Table 5 and Fig. 5, with the optimal feature subset generated by the proposed feature selection method, the sensitivity, specificity, accuracy, MCC, and AUC of the prediction model are 0.776, 0.888, 0.832, 0.668, and 0.872, respectively, better than those of the prediction model using all features. Therefore, the Relief combine with IFS is effective to eliminate irrelevant and redundant features existing in the BpB feature space. The final anti-angiogenic peptide prediction model will be constructed by the ensemble classifier NB + LR combined with the proposed feature selection method.

**Table 5:** Prediction results with the proposed feature selection method or not.

Method	Sn	Sp	Acc	MCC	AUC
Without feature selection	0.766	0.879	0.822	0.649	0.870
With feature selection	0.776	0.888	0.832	0.668	0.872

### Performance Comparisons with the Existing Method on Benchmark Dataset

To objectively access the prediction ability for anti-angiogenic peptide prediction, performance measures obtained by our method and the existing method^24^ on the same benchmark dataset are compared. The detailed prediction results based on 10-fold cross validation are listed in Table 6. As given in Table 6, the proposed method achieves ideal results, obviously outperforming the previous study^24^. More specifically, the specificity, accuracy, and MCC of the proposed method are significantly (i.e., approximately 0.150, 0.084, 0.168) higher than those of the existing method^24^. Therefore, the proposed ensemble method is effective in predicting anti-angiogenic peptides, which may provide a deeper understanding for the essential angiogenic homeostasis, thereby beneficial to develop antineoplastic therapies.

**Table 6:** Performance comparisons with the existing method on benchmark dataset.

Method	Sn	Sp	Acc	MCC	AUC
Ref.^24^	0.757	0.738	0.748	0.50	—
This study	0.776	0.888	0.832	0.668	0.872

The outstanding performance of our predictor is mainly attributed to 3 aspects. (1) The BpB features contain discriminative information for distinguish anti-angiogenic pepetides from non-anti-angiogenic pepetides. (2) The Relief combined with IFS can make a distinct contribution to selecting the optimal features for identifying anti-angiogenic pepetides. (3) The ensemble learning method proposed here takes advantage of superiorities of individual classifiers with respect to specific data structure and distribution.

For classification problems, numerous studies have demonstrated that an effective way to improve prediction performance is to design an advanced learning algorithm. Based on laplacian regularized sparse subspace learning, extreme gradient boosting machine, and ensemble learning, respectively, the computational models developed by Chen X *et al*. achieved superior prediction accuracy for miRNA-disease association^29–31^. Based on ensemble rotation forest learning, Wang L *et al*. proposed an effective computation method for large-scale identification of protein-protein interactions^32^. Based on ensemble learning, a new sequence-based method proposed by Li JQ *et al*. shows a good performance for self-interacting protein prediction^33^. These existing learning algorithms will inspires us to propose novel machine learning models or other ensemble models to identify anti-angiogenic peptides in the future work.

## Materials and Methods

### Benchmark Dataset

In order to objectively make comparisons with the previous study for anti-angiogenic peptide prediction, the benchmark dataset constructed by Ettayapuram Ramaprasad AS *et al*.^24^ containing 107 positive and 107 negative samples is employed to construct the proposed prediction model. None of the peptides has 70% sequence identity to any other in the positive samples. For detailed information of the benchmark dataset, please refer to Table S3.

### Feature Extraction

The selection of appropriate protein feature representation methods that can truly reflect their intrinsic correlation with the attribute to be predicted is critical to establish a powerful protein attribute predictor^34^. Appropriate feature representations make it easier for the classifier to recognize underlying regularities, which is vital to the success of classifier learning^35^. Generally, one single feature extraction method cannot capture enough discriminative information for protein attribute predictions. Multiple features from different sources can complement each other in enhancing the discrimination power of a hypothesis. It is an extremely difficult task to discover the best combination of features that are distinctively responsible for accurate classification as no standard technique is available for it^36^. In this study, after investigating the sequence properties of anti-angiogenic peptides carefully, hybrid features extracted from CTD, BpB, and DFT, which are all correlated with the intrinsic properties of these peptides, are adopted for anti-angiogenic peptide identification.

#### Bi-profile Bayes

Statistical differences between positive sample set and negative sample set exist in the frequencies of 20 native amino acids occurred along peptide sequences, i.e. Cys, Pro, Ser, Arg, Trp, Thr and Gly are predominant in anti-angiogenic peptides while Ala, Asp, Ile, Leu, Val and Phe are not preferred in these peptides^24^. Important single peptides of a protein are usually hidden at its N- or C-terminal region, which is considered as a key factor for protein function determination^37^. As demonstrated in preliminary analysis^24^, there are statistical differences about the position-specific amino acid composition between positive and negative samples at the N-terminal region and C-terminal region. Certain residues (Ser, Pro, Trp, Thr, Arg, and Cys) are preferred at various positions at the N-terminal region of anti-angiogenic peptides while Ala, Val, Glu, Met, Phe, and Asn are prominent at various positions at the N-terminal region of non-anti-angiogenic peptides. For anti-angiogenic peptides, Cys, Gly, Asp, Ser, and Arg are prominent at different positions of the C-terminal region while Ala, Leu, Trp, Ile, and Val are preferred at distinct positions at the C-terminal region of non-anti-angiogenic peptides. In this study, BpB^38^ is utilized to calculate statistically significant differences in the distribution of amino acid residues at the N-terminal region and C-terminal region between positive and negative datasets.

Given a peptide segment *P* = {*n*_1_, …, *n*_*i*_, …, *n*_*m*_, *c*_1_, …, *c*_*i*_, …, *c*_*m*_} with *m* amino acids at the N-terminus and *m* amino acids at the C-terminus, where *n*_*i*_ is the *i*th residue at the N-terminus and *c*_*i*_ represents the *i*th residue at the C-terminus. After calculating the posterior probabilities of 20 natural amino acids at each position of the C-terminus and N-terminus from the benchmark dataset, a peptide sample can be formulated as

1 $$({P}_{N}^{1},{P}_{N}^{2},\ldots ,{P}_{N}^{m};{P}_{C}^{1},{P}_{C}^{2},\ldots ,{P}_{C}^{m};{P}_{N}^{m+1},{P}_{N}^{m+2},\ldots ,{P}_{N}^{2m};{P}_{C}^{m+1},{P}_{C}^{m+2},\ldots ,{P}_{C}^{2m}),$$

where $({P}_{N}^{1},{P}_{N}^{2},\ldots ,{P}_{N}^{m})$ and $({P}_{C}^{1},{P}_{C}^{2},\ldots ,{P}_{C}^{m})$ denote the posterior probabilities of the corresponding amino acids at each position of the N-terminus and C-terminus compared to the positive dataset, respectively.

Similarly, $({P}_{N}^{m+1},{P}_{N}^{m+2},\ldots ,{P}_{N}^{2m})$ and $({P}_{C}^{m+1},{P}_{C}^{m+2},\ldots ,{P}_{C}^{2m})$ represent the posterior probabilities of each amino acid at each position of the N-terminus and C-terminus compared to the negative dataset, respectively. The length of N-terminus or C-terminus is set as 10, then each sample is converted into a 40-dimensional feature vector.

#### Composition, Transition, and Distribution

Primary analysis based on the amino acid composition and residue propensities in the existing method^24^ reveals that certain residues (Cys, Trp, Ser, Arg, and Pro) are preferred in anti-angiogenic peptides^24^. In addition, research results in^39^ have demonstrated that anti-angiogenic peptides, for the most part, are compositionally similar and they have a relatively high incidence of hydrophobic and cationic residues. In view of the essential physicochemical properties of anti-angiogenic peptides mentioned above, 20 natural amino acids are divided into four groups on the basis of their hydrophobicity and charged character, that is the hydrophobic group *C*_1_ = {*A*, *F*, *G*, *I*, *L*, *M*, *P*, *V*, *W*}, the polar group *C*_2_ = {*C*, *N*, *Q*, *S*, *T*, *Y*}, the positively charged group *C*_3_ = {*H*, *K*, *R*}, and the negatively charged group *C*_4_ = {*D*, *E*}^40^. Based on the four groups, the concept of CTD proposed d by Dubchak I *et al*.^41^ is introduced to extract information on global composition, physicochemical property, and sequence order from peptide sequences.

With a particular property, composition (*C*) calculates the frequencies of each group in a given peptide, which is defined as

2 $$(\frac{{N}_{1}}{L},\frac{{N}_{2}}{L},\frac{{N}_{3}}{L},\frac{{N}_{4}}{L}),$$

where *N*_*i*_, *i* ∈ {1, 2, 3, 4} is the number of each group and *L* is the length of the peptide.

In a given peptide, transition (*T*) describes the frequencies of an amino acid with a particular property followed by an amino acid with another property, which is formulated by the following equation.

3 $$\frac{{N}_{i,j}+{N}_{j,i}}{L-1},$$

where *i*, *j* ∈ {1, 2, 3, 4} represents the corresponding group. *N*_*i*,*j*_ is the number of the dipeptide containing two residues from two different groups.

Distribution (*D*) expresses the distribution pattern of each group which is measured by the position of the first, 25%, 50%, 75%, and 100% of each of the four groups along the sequence, which can be calculated by

4 $$(\frac{{N}_{1,1}}{L},\ldots ,\frac{{N}_{1,5}}{L},\frac{{N}_{2,1}}{L},\ldots ,\frac{{N}_{2,5}}{L},\ldots ,\frac{{N}_{4,1}}{L},\ldots ,\frac{{N}_{4,5}}{L}),$$

where *N*_*i*,1_ is the chain length within which the first amino acid of the *i*th group is located. *N*_*i*,2_, *N*_*i*,3_, *N*_*i*,4_, *N*_*i*,5_ measure the chain lengths within which the 25%, 50%, 75%, and 100% of the amino acids of the *i*th group are located, respectively.

#### Discrete Fourier Transform

Physicochemical properties of amino acids are the most important features for protein biochemical reactions, which have a deep influence on protein structure and function forming^42^. Dings RP *et al*.^39^ have reported that hydrophobic residues are prone to occur in anti-angiogenic peptides. In addition, a protein sequence occasionally shows periodicity of hydrophobicity and hydrophilicity, which can contribute to protein attribute predictions^43^.

In this study, based on the hydrophobicity and hydrophilicity, a peptide sequence is transformed into a numerical feature vector. Then the frequency information reflecting the periodicity is merged into a set of discrete components by transferring the coded sequence to its corresponding frequency domain, which reflects the distribution of power contained in a peptide sequence over the frequencies^44^. Via the transformation, some important features hidden in the sequence could be revealed without information loss. This goal can be achieved with the help of DFT. DFT^45^, a transformation approach converting numerical values into frequency domain, reveals periodicities of input data as well as the relative strengths of various periodic components.

The DFT of a given peptide sequence with the length of *L* is defined as

5 $$F(k)=\sum _{n=1}^{L}\,H({p}_{n}){e}^{-2\pi nkj/L},\,k=0,1,\ldots ,L-1,$$

where *F*(*k*) represents the periodicity characteristics of the sequence and the compositional patterns by sinusoidal waves with various frequencies. *H*(*p*_*n*_), *n* = 0, 1, …, *L* − 1 denotes physicochemical property values of each amino acid of the given peptide sequence.

The DFT power spectrum at frequency *k* is defined as

6 $$PS(k)=|F(k){|}^{2},k=0,1,\ldots ,L-1.$$

The fourier coefficients partially reflect the sequence order information. Generally, the low-frequency components of DFT contain more biological significance than high frequency noisy ones^46^. Hence the DFT power spectrums at low frequencies are chosen as effective features. The minimum length of peptide sequences in the benchmark dataset is 10. For the hydrophobicity or hydrophilicity of amino acids, we use 10 low frequency DFT power spectrums to represent the sequences.

### Feature Selection

Not all the extracted features can contribute to the prediction accuracy. Commonly, hybrid features from various sources would bring some redundant or irrelevant features, which may deteriorate the generalization ability and the performance of learning algorithms^25^. To eliminate the redundant features and improve prediction performance, it is necessary to develop feature selection techniques to pick out the optimal features, which can also contribute to simplifying the classifier and gaining deeper insights into the intrinsic properties of protein sequences. To obtain the optimal feature subset, the Relief algorithm combined with IFS method is employed in this study.

In order to describe the correlation between class labels and features, Kira K and Rendell LA developed a feature selection algorithm called Relief in 1992^47^. It runs in low-order polynomial time, and is noise-tolerant to feature interactions. With the ability to differentiate the class labels of close samples, Relief is an effective iterative algorithm to evaluate the prediction ability of each feature by setting feature weights within the interval $[0,\,1]$^48^, which is represented as

7 $${W}_{p}^{i+1}={W}_{p}^{i}-\frac{diff(Y,{s}_{i},NH({s}_{i}))}{n}+\frac{diff(S,{s}_{i},NM({s}_{i}))}{n},$$

8 $$diff(\ast ,x,y)=\{\begin{array}{ll}\parallel x-y\parallel , & x\ne y\\ 0, & x=y\end{array},$$

where ${W}_{p}^{i}$ and ${W}_{p}^{i+1}$ stands for the assigned weights for a given feature *p* at the *i*th iteration and the (*i* + 1)th iteration, respectively. *s*_*i*_ denotes one of the samples in the dataset numbered *i*. *NH*(*s*_*i*_), called nearest hit, denotes the neighbor samples of *s*_*i*_ in the sample set *Y* where the samples have the same class label as *s*_*i*_. *NM*(*s*_*i*_), called nearest miss, denotes the neighbor samples of *s*_*i*_ in the sample set *S* where the samples have the different class labels as *s*_*i*_. *n* denotes the number of samples generated randomly. In order to search the nearest neighbor sample, this paper uses the formula (8) to calculate the distance of different samples.

In general, the feature weight calculated by the Relief algorithm is positively correlated with the prediction ability of the corresponding feature. According to the weights from highest to lowest, the considered features can be sorted.

In order to obtain the optimal feature subset, the IFS (Incremental Feature Selection) method, a proverbial searching strategies in feature selection, is adopted in this study. Based on the feature ranking, the IFS method is implemented in the following steps: First, generate an empty feature subset, and then add the features to the feature subset one by one with the weight from highest to lowest. At each iteration, with a new feature added, a new feature subset is generated to construct a new prediction model. The feature subset that achieves the highest prediction accuracy will be selected as the optimal feature subset.

### Machine Learning Method

#### Random Forest

The random forest (RF) algorithm, proposed by Breiman^49^, is a supervised learning algorithm that has been successfully employed in classification problems^50,51^ and achieves satisfactory performance. It is an ensemble classifier generating a multitude of decision trees, where each decision tree is constructed based on the benchmark dataset and produces a classification label. To predict a test sample, its feature vector is put into each of the decision trees in the forest, and each tree gives a vote suggesting one class. The predicted result of the RF is decided based on the most votes given by all the individual trees^52^. RF can reduce the output variance of individual trees, and thus improves the stability and accuracy of classification. In addition, it is relatively robust to noise and outliers^49^.

#### Radial Basis Function Network

The radial basis function network (RBFNetwork) is suitable for solving function approximation and pattern classification problems due to its faster training procedure and better approximation capabilities^53^. In the classical RBFNetwork, there is an input layer, a hidden layer with a non-linear RBF activation function, and a linear output layer^54^. It uses the k-means clustering to provide the basis functions. The basis functions are usually chosen as Gaussian and the number of hidden units are fixed a priori using some properties of input data. RBFNetworks have advantages of strong tolerance to noise and good generalization^55^.

#### Naïve Bayes

Naïve bayes (NB) is generally known as a simple probabilistic classifier, which has been successfully used in the realm of bioinformatics^56,57^. The naïve bayes assumes the attribute variables to be independent from each other, which can greatly reduce the complexity of the development of the classifier.

#### Logistic Regression

The crucial limitation of linear regression is that it cannot deal with dependent variables that are dichotomous and categorical. Logistic regression (LR) is an effective method to find the best fitting model to describe the relationship between the categorical dependent variable and a set of independent numeric variables^58^.

#### Nearest Neighbor Algorithm

Nearest neighbor algorithm (NNA) is a machine learning technique based on cluster theory. Despite its simplicity, NNA often performs nearly as well as more sophisticated methods. Based on the NNA classification principle, a new sample is assigned to the same class as the one in the benchmark dataset that is nearest to the query sample^59^.

### Classifier Fusion

Every single learning strategy has its own shortcomings and could not always perform well on all datasets^60^. The classifier fusion emerges as a promising measure to overcome this problem^28,61^. A fusion of classifiers is a collection of multiple basic individual classifiers with diverse learning policies and then aggregates the outputs of all independent classifiers to tackle the same classification task^62^. In general, the outputs of different single classifiers tend to be different for a given classification problem. But at the same time they have the ability to correct each other’s mistakes. Therefore, the prediction ability of classifier fusion is usually superior to that of its component single classifier^63^. Hansen LK and Salamon P^64^ has theoretically demonstrated that the classifier fusion gives much better performance compared to its base classifiers.

In this study, we evaluate prediction performance of different classifiers including radial basis function network, naïve bayes, logistic regression, nearest neighbor algorithm and random forest, respectively. Then the ultimate result is determined by the average probability of the outputs obtained from one classifier which is good at predicting negative class (with a higher specificity) and another one which is good at predicting positive class (with a higher sensitivity). WEKA machine learning platform^65^ is used for implementing all the algorithms and the classifier fusion method.

### Performance Evaluation

Independent dataset test, jackknife test, and sub-sampling test are the 3 common methods to measure the performance of a predictor^66^. For a given prediction problem, the output result generated by the jackknife test is unique while the other 2 methods are not^67,68^. Therefore, the jackknife test can obtain a more strict and objective prediction result, which make it extensively applied to verify the performance of prediction models^27,69^. For the purpose of reducing the complexity of computing, 10-fold cross validation test^24^, one of sub-sampling test, is used to measure the performance of the anti-angiogenic peptide predictors.

Based on the prediction result generated by the 10-fold cross validation test, the following evaluation indexes are calculated to compare the proposed method with the existing method.

Sensitivity (*Sn*) represents the prediction accuracy of anti-angiogenic peptides, which is expressed as:

9 $${S}_{n}=\frac{TP}{TP+FN},$$

Specificity (*Sp*) represents the prediction accuracy of non-anti-angiogenic peptides, which is given by:

10 $${S}_{p}=\frac{TN}{TN+FP},$$

Accuracy (*Acc*) represents the overall prediction accuracy of all samples in the dataset, which is defined as:

11 $$Acc=\frac{TP+TN}{TP+FP+TN+FN},$$

Matthew’s correlation coefficient (MCC)^70^ is another effective measure for performance evaluation and calculated as:

12 $$MCC=\frac{TP\ast TN-FP\ast FN}{\sqrt{(TP+FN)\,(TP+FP)\,(TN+FP)\,(TN+FN)}},$$

where *TP*, *TN*, *FP*, and *FN* denote number of correctly predicted anti-angiogenic peptides, number of correctly predicted non-anti-angiogenic peptides, number of non-anti-angiogenic peptides incorrectly predicted as anti-angiogenic peptides, and numer of anti-angiogenic peptides incorrectly predicted as non-anti-angiogenic peptides, respectively.

To provide more insight into the prediction performance for anti-angiogenic peptides, the receiver operating characteristic (ROC) curve^71^ is plotted, and the area under the ROC curve (AUC) is calculated. The prediction model with a higher AUC value indicates that it achieves a better prediction performance^49^.

## Conclusions

Anti-angiogenic peptides are thought to have physiological functions and excellent therapeutic potential for angiogenesis-related diseases. Identification of anti-angiogenic peptides accurately may not only contribute to better understanding essential angiogenic homeostasis within tissues, but also provide significant clues to develop antineoplastic therapies. To identify anti-angiogenic peptides, an ensemble learning method has been presented in this study by fusing an individual classifier with the best sensitivity and another classifier with the best specificity. To decrease the complexity of computation, the Relief algorithm followed by the IFS method is employed to eliminate the redundant features. Based on the benchmark dataset, the accuracy of various feature spaces (i.e., BpB, CTD, DFT) with respect to the corresponding optimal individual classifiers lies in the range of 0.636 to 0.804, indicating discriminative power of features. The accuracy, MCC, and AUC of BpB with an NB classifier are 0.804, 0.626, and 0.902, respectively, which represents the highest prediction results among the various feature spaces, demonstrating that position-specific statistical differences at the N and C-terminal region are suitable to identify anti-angiogenic peptides. The accuracy of BpB on the ensemble classifier (i.e., NB + LR) is 0.822, revealing that an appropriate ensemble classifier can effectively improve prediction performance. In addition, by means of the Relief-IFS, the sensitivity, specificity, accuracy, MCC, and AUC of the prediction model are 0.776, 0.888, 0.832, 0.668, and 0.872, respectively, better than those of the prediction model using all features. Performance comparisons with the existing method on the same dataset indicate that the proposed ensemble method is effective in predicting anti-angiogenic peptides.

## Electronic supplementary material

Table S1

Table S2

Table S3
