# Supplementary material for: Using a Classifier Fusion Strategy to Identify Anti-angiogenic Peptides
Source: Sci Rep. 2018 Sep 14;8:14062. doi: 10.1038/s41598-018-32443-w (PMC6138733; doi:10.1038/s41598-018-32443-w)
Supplement: Supplementary file 1 — Table S1 [file 41598_2018_32443_MOESM1_ESM.pdf]

Using a Classifier Fusion Strategy to Identify Anti-angiogenic Peptides

Lina Zhang[1], Runtao Yang[1,*], and Chengjin Zhang[1]

[1]School of Mechanical, Electrical and Information Engineering, Shandong University at Weihai, Weihai, 264209, China
[*]Corresponding Author (Email: yrt@sdu.edu.cn)

Table S1: The feature ranking in the Bi-profile Bayes (BpB) feature space given by the Relief algorithm		
**Index**	**Feature_Name**	**Weight**
1	bpb_6	0.093894
2	bpb_3	0.083326
3	bpb_10	0.07566
4	bpb_30	0.073972
5	bpb_16	0.055675
6	bpb_11	0.055443
7	bpb_14	0.048618
8	bpb_20	0.047928
9	bpb_2	0.045819
10	bpb_23	0.041768
11	bpb_38	0.041121
12	bpb_19	0.033469
13	bpb_34	0.032944
14	bpb_36	0.031963
15	bpb_13	0.029855
16	bpb_40	0.029634
17	bpb_26	0.028325
18	bpb_27	0.027103
19	bpb_7	0.025823
20	bpb_4	0.025578
21	bpb_12	0.025578
22	bpb_1	0.02465
23	bpb_25	0.023318
24	bpb_18	0.02209
25	bpb_32	0.020461
26	bpb_21	0.020094
27	bpb_22	0.019626
28	bpb_17	0.017227
29	bpb_33	0.015654
30	bpb_31	0.015327
31	bpb_29	0.015187
32	bpb_24	0.014299
33	bpb_9	0.014206
34	bpb_39	0.010514
35	bpb_5	0.009346
36	bpb_28	0.006869
37	bpb_35	0.002444
38	bpb_8	0.001454
39	bpb_15	-0.005607
40	bpb_37	-0.009872
