# Supplementary material for: Using a Classifier Fusion Strategy to Identify Anti-angiogenic Peptides
Source: Sci Rep. 2018 Sep 14;8:14062. doi: 10.1038/s41598-018-32443-w (PMC6138733; doi:10.1038/s41598-018-32443-w)
Supplement: Supplementary file 2 — Table S2 [file 41598_2018_32443_MOESM2_ESM.pdf]

Using a Classifier Fusion Strategy to Identify Anti-angiogenic Peptides

Lina Zhang[1], Runtao Yang[1,*], and Chengjin Zhang[1]

[1]School of Mechanical, Electrical and Information Engineering, Shandong University at Weihai, Weihai, 264209, China
[*]Corresponding Author (Email: yrt@sdu.edu.cn)

Table S2: The detailed prediction results of the prediction model at each iteration in the incremental feature selection (IFS) procedure									
**Id**	**FP**	**FN**	**TN**	**TP**	**Sensitivity**	**Specificity**	**Accuracy**	**MCC**	**AUC**
1	3	77	104	30	0.280374	0.971963	0.626168	0.349355	0.681719
2	11	63	96	44	0.411215	0.897196	0.654206	0.352886	0.72312
3	9	67	98	40	0.373832	0.915888	0.64486	0.344764	0.729671
4	10	65	97	42	0.392523	0.906542	0.649533	0.348651	0.741069
5	14	57	93	50	0.46729	0.869159	0.668224	0.367423	0.778758
6	13	59	94	48	0.448598	0.878505	0.663551	0.362291	0.770286
7	16	57	91	50	0.46729	0.850467	0.658879	0.344014	0.767578
8	17	59	90	48	0.448598	0.841121	0.64486	0.315001	0.767665
9	18	53	89	54	0.504673	0.831776	0.668224	0.356034	0.778409
10	17	54	90	53	0.495327	0.841121	0.668224	0.358569	0.78889
11	17	44	90	63	0.588785	0.841121	0.714953	0.444284	0.811687
12	17	44	90	63	0.588785	0.841121	0.714953	0.444284	0.813608
13	17	45	90	62	0.579439	0.841121	0.71028	0.435745	0.81981
14	17	43	90	64	0.598131	0.841121	0.719626	0.452824	0.825312
15	16	40	91	67	0.626168	0.850467	0.738318	0.489098	0.832999
16	16	43	91	64	0.598131	0.850467	0.724299	0.4636	0.832824
17	15	41	92	66	0.616822	0.859813	0.738318	0.491362	0.833872
18	17	41	90	66	0.616822	0.841121	0.728972	0.469917	0.830466
19	16	41	91	66	0.616822	0.850467	0.733645	0.480592	0.824351
20	15	41	92	66	0.616822	0.859813	0.738318	0.491362	0.823041
21	14	41	93	66	0.616822	0.869159	0.742991	0.502234	0.821294
22	14	42	93	65	0.607477	0.869159	0.738318	0.493844	0.822867
23	14	44	93	63	0.588785	0.869159	0.728972	0.477079	0.833348
24	15	39	92	68	0.635514	0.859813	0.747664	0.508278	0.83195
25	14	39	93	68	0.635514	0.869159	0.752336	0.519039	0.8392
26	13	39	94	68	0.635514	0.878505	0.757009	0.529901	0.851341
27	12	38	95	69	0.64486	0.88785	0.766355	0.54917	0.856319
28	14	32	93	75	0.700935	0.869159	0.785047	0.578335	0.866451
29	15	32	92	75	0.700935	0.859813	0.780374	0.567962	0.863656
30	15	32	92	75	0.700935	0.859813	0.780374	0.567962	0.861123
31	15	34	92	73	0.682243	0.859813	0.771028	0.550809	0.866888
32	16	33	91	74	0.691589	0.850467	0.771028	0.54903	0.862433
33	16	32	91	75	0.700935	0.850467	0.775701	0.557672	0.862957
34	14	29	93	78	0.728972	0.869159	0.799065	0.604096	0.864879
35	14	27	93	80	0.747664	0.869159	0.808411	0.621426	0.862957
36	14	26	93	81	0.757009	0.869159	0.813084	0.630144	0.865141
37	15	26	92	81	0.757009	0.859813	0.808411	0.620108	0.866975
38	13	24	94	83	0.775701	0.878505	0.827103	0.65769	0.873526
39	12	24	95	83	0.775701	0.88785	**0.831776**	0.667764	0.872303
40	13	25	94	82	0.766355	0.878505	0.82243	0.648954	0.869508
