# Supplementary material for: Using a Classifier Fusion Strategy to Identify Anti-angiogenic Peptides
Source: Sci Rep. 2018 Sep 14;8:14062. doi: 10.1038/s41598-018-32443-w (PMC6138733; doi:10.1038/s41598-018-32443-w)
Supplement: Supplementary file 3 — Table S3 [file 41598_2018_32443_MOESM3_ESM.pdf]

Using a Classifier Fusion Strategy to Identify Anti-angiogenic Peptides

Lina Zhang[1], Runtao Yang[1,*], and Chengjin Zhang[1]

[1]School of Mechanical, Electrical and Information Engineering, Shandong University at Weihai, Weihai, 264209, China
[*]Corresponding Author (Email: yrt@sdu.edu.cn)

Table S3: The benchmark dataset with 107 anti-angiogenic peptides and non-anti-angiogenic peptides		
**Id**	**Sequence**	**Class**
1	AAPFLECQGRQGTCHFFAN	anti-angiogenic peptides
2	ANIKLSVQMKLFKRHLKWKIIVKLNDGRELSLDA	anti-angiogenic peptides
3	CDSDSDITWDQLWDLMK	anti-angiogenic peptides
4	CETWRTETTGATGQASSLLSGRLLEQKAASCHNSYIVLCIENSFMTSFSK	anti-angiogenic peptides
5	CKITRCPMIPCYISSPDECLWMDWVTEKNINGHQAKFFACIKRSDGSCAWYRGAAPPKQEFLDIEDP	anti-angiogenic peptides
6	CQNHHAKHGKVC	anti-angiogenic peptides
7	DDDDDNDKIPDDRDN	anti-angiogenic peptides
8	DDDDKRAGSPSGGPFCALARQPLTGSPPNERAFFCSSRDV	anti-angiogenic peptides
9	DGRELCLDPKENWVQRVVEKFLK	anti-angiogenic peptides
10	DGRKICLDPDAPRIKKIVQKKL	anti-angiogenic peptides
11	DLWIRETLTSPKSLTG	anti-angiogenic peptides
12	DPFFKVPVNKLAAAVSNFGYDLYRVRSSTSPTTN	anti-angiogenic peptides
13	DPPEGLUGTKPPROH	anti-angiogenic peptides
14	DRSTREPIYMSTI	anti-angiogenic peptides
15	DSSPVSTEQLAPTA	anti-angiogenic peptides
16	EGLPGPQGPKGFPGLPGLTG	anti-angiogenic peptides
17	EIPSCESSASPDQSDSSVPPEE	anti-angiogenic peptides
18	EKSSRPEFYKVILGAHEEYIRG	anti-angiogenic peptides
19	EKYEGKISKTMSGLDCQAWDS	anti-angiogenic peptides
20	ESLARPCAPGAPAEARL	anti-angiogenic peptides
21	FCNINNVCNFASRNDYSYW	anti-angiogenic peptides
22	FLKDHRISTFKNWPF	anti-angiogenic peptides
23	FLSSRLQDLYSIVRRADRAA	anti-angiogenic peptides
24	GFHDHGPCDPPSHK	anti-angiogenic peptides
25	GHRATSDLASTGEESQD	anti-angiogenic peptides
26	GPWEDCSVSCGGGEQLRSR	anti-angiogenic peptides
27	GPWEPCSVTCSKGTRTRRR	anti-angiogenic peptides
28	GPWERCTAQCGGGIQARRR	anti-angiogenic peptides
29	GPWGDCSRTCGGGVQFSSR	anti-angiogenic peptides
30	GPWGPCSGSCGPGRRLRRR	anti-angiogenic peptides
31	HGLGHGHEQQHGLGHGHKFKLDDDLEHQGGHVLD	anti-angiogenic peptides
32	HGSTTLRDITV	anti-angiogenic peptides
33	HHPHGHHPHGHHPHGHHPHG	anti-angiogenic peptides
34	HKLINTEGHHS	anti-angiogenic peptides
35	HTHQDFQPVLHLVALNTPLSGGMRGIR	anti-angiogenic peptides
36	IMRIKQGQIGQMTI	anti-angiogenic peptides
37	INEFLERSGIPRQRNQ	anti-angiogenic peptides
38	INGSLDKRLLPDVET	anti-angiogenic peptides
39	INGSLDKRVQDCYHG	anti-angiogenic peptides
40	INLEACLKRGRT	anti-angiogenic peptides
41	ITMQGIQGQKIRMIMF	anti-angiogenic peptides
42	KAFDITYVRLKF	anti-angiogenic peptides
43	KCGHKHQCAVHN	anti-angiogenic peptides
44	KIKSCYYLPCFVTS	anti-angiogenic peptides
45	KRFKQDGGWSHWSPWSSCSVTCGDGVITRIRLCNSPSPQMNGKPCEGEARETKACKKDACPI	anti-angiogenic peptides
46	KSVRGKGKGQKRKRKKSRYK	anti-angiogenic peptides
47	LHCPALVTYNTDTFESMPNPEGRYTFGASCV	anti-angiogenic peptides
48	LLRISLLLIQSWLE	anti-angiogenic peptides
49	LPGLTGSKGVRGISGLPGFSG	anti-angiogenic peptides
50	LRRFSTMPFMFCNINNVCNF	anti-angiogenic peptides
51	LRSRGELVAKFLAGEQSPEDYVAE	anti-angiogenic peptides
52	LSSTCILVLVKDILVLVVKEILVLVVKDKPI	anti-angiogenic peptides
53	LVPLPKIKNSTFT	anti-angiogenic peptides
54	LVPRGSRAGSPSGGPFCALARQPLTGARLMSGLFFALHET	anti-angiogenic peptides
55	MEPECNLNCTD	anti-angiogenic peptides
56	MFSPILSLEIILALATLQSVFAQPVICTTVGSAAEGS	anti-angiogenic peptides

57	MLQNSAVLLLLVISASA	anti-angiogenic peptides
58	NGKQVCLDPEAPFLKKVIQKILDS	anti-angiogenic peptides
59	NGREACLDPEAPMVQKIVQKMLKG	anti-angiogenic peptides
60	NGRKACLNPASPIVKKIIEKMLNS	anti-angiogenic peptides
61	NVLLSPLSVATALSALSLGAEQRTES	anti-angiogenic peptides
62	PGLKGKRGDSGSPATWTTRG	anti-angiogenic peptides
63	PTGERLRTCERLSYP	anti-angiogenic peptides
64	QEPHRHSIFTPQTNPRADLEKN	anti-angiogenic peptides
65	QMIVIELGTNPLKSSGIENGAFQGMK	anti-angiogenic peptides
66	QPWGTCSESCGKGTQTRAR	anti-angiogenic peptides
67	QPWSQCSATCGDGVRERRR	anti-angiogenic peptides
68	RCRLAERRQIAK	anti-angiogenic peptides
69	RIFGESVSLRVQDWEW	anti-angiogenic peptides
70	RPFVEMYSEIPE	anti-angiogenic peptides
71	RQVFQVAYIIIKA	anti-angiogenic peptides
72	RRPAAAGKRRREKQRPSDKPRR	anti-angiogenic peptides
73	RRPKGRAMRREKQRPSDKPRR	anti-angiogenic peptides
74	RRPKGRGKRRREKQRPTDCHLCGDAVPRR	anti-angiogenic peptides
75	SAWRACSVTCGKGIQKRSR	anti-angiogenic peptides
76	SEWSDCSVTCGKGMRTRQR	anti-angiogenic peptides
77	SKRKSRPVSVKTFEDIPLEEP	anti-angiogenic peptides
78	SKWSECSRTCGGGVKFQER	anti-angiogenic peptides
79	SPNITVTLKKFPL	anti-angiogenic peptides
80	SPSTHPNEGLEENYCRNPDN	anti-angiogenic peptides
81	SPWSKCSAACGQTGVQTRTR	anti-angiogenic peptides
82	SPWSPCSGNCSTGKQQRTR	anti-angiogenic peptides
83	SPWSPCSTSCGLGVSTRI	anti-angiogenic peptides
84	SPWSQCTASCGGGVQTR	anti-angiogenic peptides
85	SPWTKCSATCGGGHYMRTR	anti-angiogenic peptides
86	SRTVRKTSRLWSSLSLNTCNNVHSKS	anti-angiogenic peptides
87	SSTSPHRPRFS	anti-angiogenic peptides
88	TAWGPCSTTCGLGMATRV	anti-angiogenic peptides
89	TEWSACNVRCGRGWQKRSR	anti-angiogenic peptides
90	TEWSVCNSRCGRGYQKRTR	anti-angiogenic peptides
91	TEWTACSKSCGMGFSTRV	anti-angiogenic peptides
92	TGASSEEEDPF	anti-angiogenic peptides
93	TKPPRKRPPKTKKRPPKTTKPPRGZOG	anti-angiogenic peptides
94	TKWTPCSRTCGMGISNRV	anti-angiogenic peptides
95	TLPFAYCNIHQVCHYAQRNDRSYWL	anti-angiogenic peptides
96	TQWTSCSKTCNSGTQSRHR	anti-angiogenic peptides
97	TSLDASIIWAMMQN	anti-angiogenic peptides
98	TSWSQCSKTCGTGISTRV	anti-angiogenic peptides
99	TTITGKKCQSWAAMFPHRHSKT	anti-angiogenic peptides
100	VGSGGCMFGNGK	anti-angiogenic peptides
101	VIFEWTLLQVLSESDQDQSLEVFLT	anti-angiogenic peptides
102	VVGSPSAQDEASPL	anti-angiogenic peptides
103	WTRCSSSCGRGVSVRSR	anti-angiogenic peptides
104	YCNINEVCHYARRNDKSYWL	anti-angiogenic peptides
105	YPYDVPDYASL	anti-angiogenic peptides
106	YRIPIVRRLQRR	anti-angiogenic peptides
107	YTMNPRKLFDY	anti-angiogenic peptides
108	ADNWQSFDRWKDH	non-anti-angiogenic peptides
109	AEALAALRALADKNQVF	non-anti-angiogenic peptides
110	AFAQFGSDLDAATQKLLNRGARLTELMKQPQ	non-anti-angiogenic peptides
111	AGAGYALLALIGTEAAS	non-anti-angiogenic peptides
112	AKAAETKSSSEQELRITQS	non-anti-angiogenic peptides
113	ATSINNSSLPDV	non-anti-angiogenic peptides
114	AVVQKRFGFPEGSV	non-anti-angiogenic peptides
115	DDVWNMKYLRGFKWADLMEQVQRE	non-anti-angiogenic peptides
116	DKAFIAFLEETFDQFLP	non-anti-angiogenic peptides
117	DMEAFTKLTDNIFLE	non-anti-angiogenic peptides

118	DVSKLKEGEQYMSFCTFPGHSALM	non-anti-angiogenic peptides
119	EALDAARYYANV	non-anti-angiogenic peptides
120	EASGPSFVSSHYLQESPGGISLEGSELTFPD	non-anti-angiogenic peptides
121	EDWSLDSRPGKSTKNSRNK	non-anti-angiogenic peptides
122	EGELLILENVRFNKGEKKDD	non-anti-angiogenic peptides
123	EHNDLRLCCKQIVEEA	non-anti-angiogenic peptides
124	EKAGLKIVAAKMLQLSQAQAEG	non-anti-angiogenic peptides
125	EKQIEQLVAQDLVRHFADLYRIDIPT	non-anti-angiogenic peptides
126	ELLIEDHIKTACNWGTTHK	non-anti-angiogenic peptides
127	ENGSTAIVVGRPITQAADPQKAYE	non-anti-angiogenic peptides
128	ERYAALLHDLGKAKTPSDILPRHHGHDLAGVEPVRKVNQRLRAPKHCAEL	non-anti-angiogenic peptides
129	FEPQVMKIMANVRPDRQTVLFSATFPRNMEALARKTLNKPVEIVVGGKSVVAPEITQIVE VR	non-anti-angiogenic peptides
130	FFTPSASHPAYVNFA	non-anti-angiogenic peptides
131	FGRLGTMFGSDLYNIKPDLV	non-anti-angiogenic peptides
132	FTSALSRAQKT	non-anti-angiogenic peptides
133	FTVRKISNGEGVERAFQTH	non-anti-angiogenic peptides
134	GALTDPTAQLVYLQKDGGL	non-anti-angiogenic peptides
135	GGPAERLTYEGDYNARGV	non-anti-angiogenic peptides
136	GHENISTTQIYTHLDFQHLADVYDQAHPRARKKSSQHKEE	non-anti-angiogenic peptides
137	GIVFQFFNLIPTLTVLENITLP	non-anti-angiogenic peptides
138	GKGVKTEFNRHVEDIKRESDGAWVL	non-anti-angiogenic peptides
139	GKSVADAIAILTFTPNKAAEII	non-anti-angiogenic peptides
140	GLHKGNKVNLTLRPAPANTGLIFRRVD	non-anti-angiogenic peptides
141	GMIFLELNFKGAEEIYYKHVHCRGGCSVFFSKISGVLTFM	non-anti-angiogenic peptides
142	GPEGMLSIAAPARDLKLATIELEHSHPLGRLWDIDVLTPEGEILSRRDYSLPPRRCLLCEQS AAVCA	non-anti-angiogenic peptides
143	GSGRTDARVHAQGQ	non-anti-angiogenic peptides
144	GSKFDSSLDRNRPFEFTLGAGQVIK	non-anti-angiogenic peptides
145	GTSIVGIVENGISVLGKIF	non-anti-angiogenic peptides
146	HGGRVTLMEITDDGLAILQFGGGCNGCSMVDFTL	non-anti-angiogenic peptides
147	HSGNIWVDSDPARKSNPRFIVLD	non-anti-angiogenic peptides
148	HSSREKIVIPFFSLLIKDIYFLNEGCA	non-anti-angiogenic peptides
149	HVLSRLSYISALGMMTRITS	non-anti-angiogenic peptides
150	HWMYQGKHVLIIFDD	non-anti-angiogenic peptides
151	IAELGTAEFPRLRIGIGRPAP	non-anti-angiogenic peptides
152	IEHPVLMARKPRFR	non-anti-angiogenic peptides
153	IEVTHWVQSRRAYAQGALEAARRLIGRPP	non-anti-angiogenic peptides
154	IFTFAGLIDHSHDFIIGFHAV	non-anti-angiogenic peptides
155	IHRAAGPALINACY	non-anti-angiogenic peptides
156	IIYITEEMGLLLGYSPIEILEKRF	non-anti-angiogenic peptides
157	INLTIAVHNGR	non-anti-angiogenic peptides
158	IVDDWIYMIEEICKI	non-anti-angiogenic peptides
159	IWIDPGFGFAKSVQQNTELLKGLDRVCQLGYPVL	non-anti-angiogenic peptides
160	KFGADCKYKFES	non-anti-angiogenic peptides
161	KLCGTNSDAYGFSANLDDS	non-anti-angiogenic peptides
162	KLLDIADLHSEMKPLH	non-anti-angiogenic peptides
163	KMVTADYIKEGA	non-anti-angiogenic peptides
164	KVSVWSKVLRSDAAWDDK	non-anti-angiogenic peptides
165	LASAYGLAKHRDGRWEWA	non-anti-angiogenic peptides
166	LGYLGPDLADSAIAVNESIIPKFLRLVDPTAAELQNF	non-anti-angiogenic peptides
167	LHRGRIPEHQREESEV	non-anti-angiogenic peptides
168	LLSEYIPSVPNCWSLLKNKKT	non-anti-angiogenic peptides
169	LMEYEQNENPMK	non-anti-angiogenic peptides
170	LVAPVTVGKGA	non-anti-angiogenic peptides
171	LVVVPPYVIRY	non-anti-angiogenic peptides
172	MASGNAVCGSSAIAAVEP	non-anti-angiogenic peptides
173	MQSLVDIAAVTELAHAAGAKV	non-anti-angiogenic peptides
174	MRIVDLGAAPGGWSQVAAKK	non-anti-angiogenic peptides
175	MTGLVKWFNPE	non-anti-angiogenic peptides
176	MVFITVSTGVGGGVVSGGKLLTGPGG	non-anti-angiogenic peptides

177	MVSSEKAMANPDSMEIDSQTISQQVLITSQSGSV	non-anti-angiogenic peptides
178	MYNSLLRMTGACHKKCVPPH	non-anti-angiogenic peptides
179	NDNTPEILYPTI	non-anti-angiogenic peptides
180	NGWLHCPADPDLIF	non-anti-angiogenic peptides
181	NITVMTSGFAFHYYVNNPH	non-anti-angiogenic peptides
182	NTKFDELMEFP	non-anti-angiogenic peptides
183	PDLCSWEEAQLSS	non-anti-angiogenic peptides
184	PKLTALVENVAEQQGINLTS	non-anti-angiogenic peptides
185	QAGADISMIGQFGVGFYSA	non-anti-angiogenic peptides
186	QAITDIHLDRV	non-anti-angiogenic peptides
187	QAQQKIILETFILFEDEVGKKL	non-anti-angiogenic peptides
188	QGCKMNNINVVYTPWANLKK	non-anti-angiogenic peptides
189	QMLEEGLLDEVQALLAAGIKGN	non-anti-angiogenic peptides
190	QTTIHVLPTAPTTVNVT	non-anti-angiogenic peptides
191	RELAAEVGSLLT	non-anti-angiogenic peptides
192	RHPDCKIVRRRGRV	non-anti-angiogenic peptides
193	RPGTPLFTVKAYL	non-anti-angiogenic peptides
194	RSERLAKLNQILRI	non-anti-angiogenic peptides
195	RVEQPENPMLDARVQAFRIA	non-anti-angiogenic peptides
196	SGNMLAGGGTLYLYALGMG	non-anti-angiogenic peptides
197	SMGPMPESGQLVFQTANLT	non-anti-angiogenic peptides
198	STDVSWEELRDTE	non-anti-angiogenic peptides
199	SYDLGERKPSSAAYQKAPT	non-anti-angiogenic peptides
200	SYRDKEMSATFRQIL	non-anti-angiogenic peptides
201	TEGIDAMGEVTIRLRRDGQLFSGHAA	non-anti-angiogenic peptides
202	TIASMPAVDEINRLSN	non-anti-angiogenic peptides
203	TLPHQRLIVATDRGIFYKM	non-anti-angiogenic peptides
204	VAATDGVGTKLKIAIDTGN	non-anti-angiogenic peptides
205	VAFKPNSTNIHVENVTVYG	non-anti-angiogenic peptides
206	VCHGNCPQSNNAFFQPLDP	non-anti-angiogenic peptides
207	VFSTTSLVVVAHYKGLTVA	non-anti-angiogenic peptides
208	VIVCLLGTAGLFLPPWLA	non-anti-angiogenic peptides
209	VKVIEAVRARTPKTT	non-anti-angiogenic peptides
210	VQDFGTALKVPK	non-anti-angiogenic peptides
211	VVRLAREPGKRESRYMH	non-anti-angiogenic peptides
212	YEDLRDESLKGLVDIGF	non-anti-angiogenic peptides
213	YFLIQSVSSTVMLLNGLYIFVN	non-anti-angiogenic peptides
214	YNLSDTIKAFSILLLTDLCI	non-anti-angiogenic peptides
